# Supplementary material for: Development of an objective index, neural activity score (NAS), reveals neural network ontogeny and treatment effects on microelectrode arrays
Source: Sci Rep. 2021 Apr 27;11:9110. doi: 10.1038/s41598-021-88675-w (PMC8079414; doi:10.1038/s41598-021-88675-w)

**Supplementary information**

Development of an objective index, neural activity score (NAS), reveals neural network ontogeny and treatment effects on microelectrode arrays

Austin P. Passaro^1,2^, Onur Aydin^3^, M. Taher A. Saif^3^, Steven L. Stice^1,2*^

^1^Regenerative Bioscience Center, University of Georgia, Athens, Georgia, United States of America

^2^Biomedical Health and Sciences Institute, Division of Neuroscience, University of Georgia, Athens, Georgia, United States of America

^3^Department of Mechanical Science and Engineering, University of Illinois at Urbana-Champaign, Urbana, IL

* Corresponding author

E-mail: [sstice@uga.edu](mailto:sstice@uga.edu)

**Supplementary Table S1. List of all MEA parameters analyzed.**

| *Parameter name* |
| --- |
| Mean firing rate (Hz) |
| Inter-spike interval (ISI) coefficient of variation – Avg |
| Number of bursting electrodes |
| Burst duration – Avg (s) |
| Number of spikes per burst |
| Mean ISI within burst – Avg |
| Median ISI within burst – Avg |
| Inter-burst (IBI) interval – Avg |
| Burst frequency (Hz) |
| Normalized burst duration IQR – Avg |
| IBI coefficient of variation – Avg |
| Burst percentage – Avg |
| Network burst frequency (Hz) |
| Network burst duration – Avg (s) |
| Number of spikes per network burst – Avg |
| Number of electrodes participating in burst – Avg |
| Number of spikes per network burst per channel – Avg |
| Network burst percentage |
| Network IBI coefficient of variation |
| Network normalized duration IQR |
| Area under normalized cross-correlation |
| Area under cross-correlation |
| Width at half height of normalized cross-correlation |
| Width at half height of cross-correlation |
| Synchrony index |

**Supplementary Table S2. EC_50_ values for all compounds analyzed in EPA network formation and toxicity assays.** Values were calculated from neural activity score, minimum individual parameter, average of all parameters, and cytotoxicity.

| *Compound* | *CASRN* | *EC_50_ (µM)* | | |
| --- | --- | --- | --- | --- |
|  |  | *Neural activity score* | *Avg. individual MEA parameter* | *Avg. cytotoxicity* |
| 1-Methyl-4-phenylpyridinium iodide | 36913-39-0 | 4.93 | 4.88 | 6.5483 |
| 1-Ethyl-3-methylimidazolium diethylphosphate | 848641-69-0 | N/A | N/A | N/A |
| 3-Iodo-2-propynyl-N-butylcarbamate | 55406-53-6 | **2.42** | 3.13 | 3.03535 |
| 6 Propyl 2 thiouracil | 51-52-5 | N/A | N/A | N/A |
| Abamectin | 71751-41-2 | **0.16** | 0.37 | 3.9339 |
| Acenaphthene | 83-32-9 | N/A | N/A | N/A |
| Aldrin | 309-00-2 | **4.14** | 4.26 | 7.30305 |
| Aspirin | 50-78-2 | N/A | N/A | N/A |
| Atrazine | 1912-24-9 | N/A | N/A | N/A |
| Auramine O | 2465-27-2 | **2.49** | 3.00 | 3.50965 |
| Benz(a)anthracene | 56-55-3 | N/A | N/A | N/A |
| Berberine chloride | 633-65-8 | **1.83** | 2.03 | 0.2499 |
| Bisphenol AF | 1478-61-1 | N/A | N/A | 16.46775 |
| Bisphenol B | 77-40-7 | N/A | N/A | N/A |
| Boric acid | 10043-35-3 | N/A | N/A | N/A |
| Boscalid | 188425-85-6 | N/A | N/A | N/A |
| Carbamic acid, butyl-, 3-iodo-2-propynyl ester | 55406-53-6 | **1.42** | 1.43 | 1.22425 |
| Chlordane | 57-74-9 | 5.86 | 5.63 | 5.7725 |
| Cloprop | 101-10-0 | N/A | N/A | N/A |
| Clove leaf oil | 8000-34-8 | N/A | N/A | N/A |
| D-Glucitol | 50-70-4 | N/A | N/A | N/A |
| Diphenhydramine hydrochloride | 147-24-0 | 18.12 | 11.56 | N/A |
| Disulfiram | 97-77-8 | 0.85 | 0.64 | 0.0954 |
| Endosulfan | 115-29-7 | **8.71** | 8.84 | 8.90985 |
| Endrin | 72-20-8 | N/A | N/A | N/A |
| Erythromycin | 114-07-8 | N/A | N/A | N/A |
| Estradiol | 50-28-2 | N/A | N/A | N/A |
| Eugenol | 97-53-0 | N/A | N/A | N/A |
| Fenamiphos | 22224-92-6 | N/A | N/A | N/A |
| Fluoxastrobin | 361377-29-9 | **0.69** | 0.89 | 1.1693 |
| Glycerol | 56-81-5 | N/A | N/A | N/A |
| Hexachlorophene | 70-30-4 | 1.92 | 1.83 | 1.8547 |
| Kepone | 143-50-0 | 5.91 | 5.39 | 8.1115 |
| L-Ascorbic acid | 50-81-7 | N/A | N/A | N/A |
| Mancozeb | 8018-01-7 | N/A | N/A | N/A |
| Manganese, tricarbonyl[(1,2,3,4,5-.eta.)-1-methyl-2,4-cyclopentadien-1-yl] | 12108-13-3 | N/A | N/A | N/A |
| Methoxychlor | 72-43-5 | **7.45** | 8.51 | 8.26275 |
| MGK 264 | 113-48-4 | N/A | N/A | N/A |
| Mirex | 2385-85-5 | **3.81** | 4.36 | 2.8233 |
| o,p'-DDT | 789-02-6 | 4.14 | 4.09 | 5.10525 |
| Parathion | 56-38-2 | N/A | N/A | N/A |
| Permethrin | 52645-53-1 | **6.16** | 7.83 | 10.45295 |
| Picoxystrobin | 117428-22-5 | **0.13** | 0.32 | 0.5449 |
| Piperonyl butoxide | 51-03-6 | N/A | N/A | 19.1251 |
| pp-DDD | 72-54-8 | 4.83 | 4.60 | 6.0773 |
| pp-DDE | 72-55-9 | 4.50 | 4.33 | 4.606 |
| pp-DDT | 50-29-3 | 4.36 | 4.33 | 4.0716 |
| Reserpine | 50-55-5 | **1.31** | 1.68 | 9.08055 |
| Rotenone | 83-79-4 | **0.41** | 0.64 | 0.03005 |
| Tamoxifen | 10540-29-1 | **3.09** | 4.35 | 6.4836 |
| Tetracycline | 60-54-8 | N/A | N/A | N/A |
| Triclosan | 3380-34-5 | **9.08** | 9.91 | 9.21815 |

N/A indicates EC50 not determined within range (0-20 µM). Individual parameter averages were only calculated if 13+ (>50%) parameters had determinable (0 < EC_50_ < 20) values. Bolded NAS values indicate compounds where NAS was more sensitive (lower EC_50_) than the avg. individual parameter value.

**Supplementary Figure S1. Coefficient of variation values for all individual parameters and neural activity score.** Values were calculated from network ontogeny data (Fig 1).


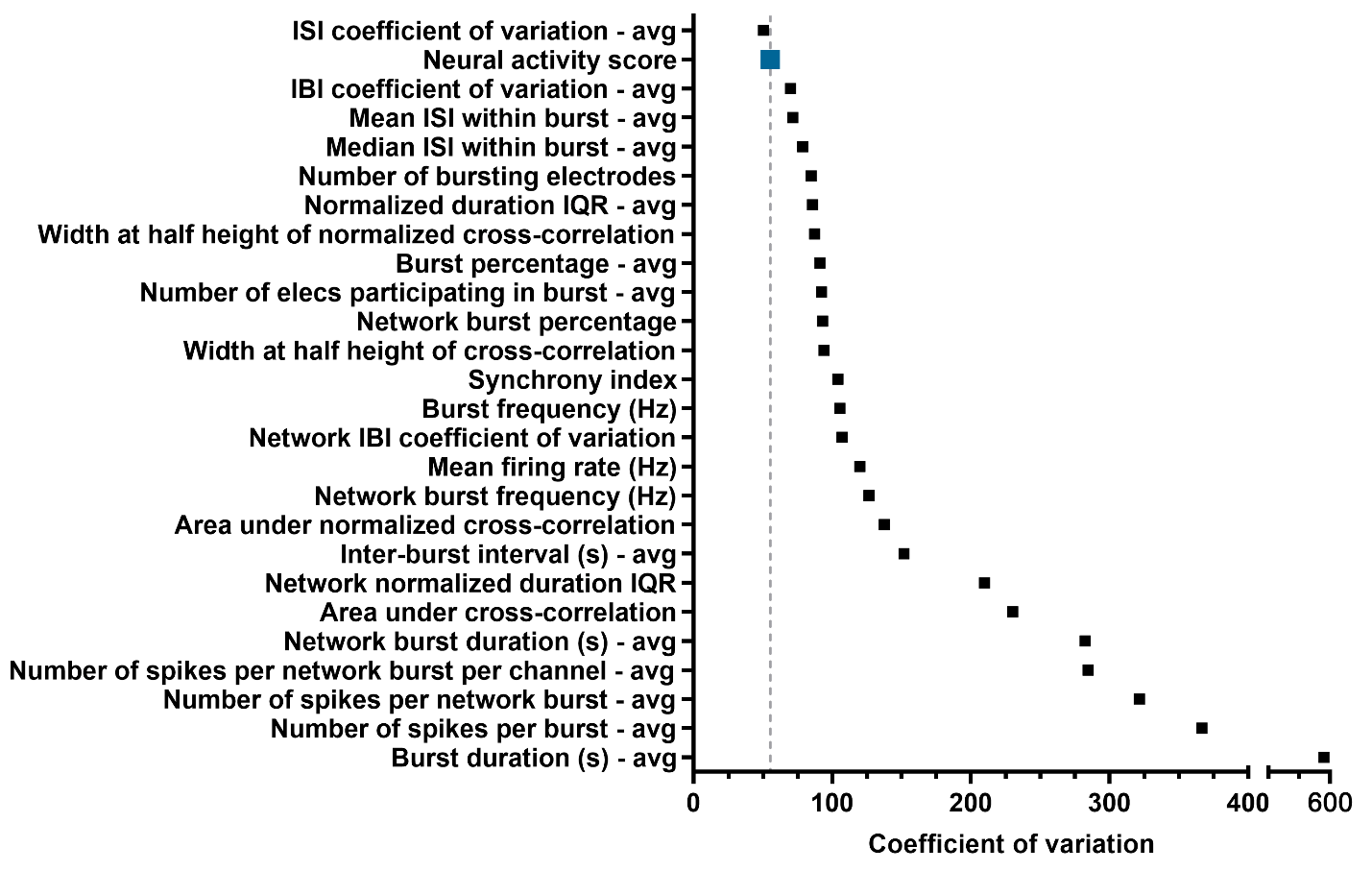

Supplement: Supplementary file 1 — Supplementary Information. [file 41598_2021_88675_MOESM1_ESM.docx]
